# A Continuous Liquid-Level Sensor for Fuel Tanks Based on Surface Plasmon Resonance

**DOI:** 10.3390/s16050724

**Published:** 2016-05-19

**Authors:** Antonio M. Pozo, Francisco Pérez-Ocón, Ovidio Rabaza

**Affiliations:** 1 Department of Optics, Faculty of Science, Edificio Mecenas, Campus Universitario de Fuentenueva, University of Granada, 18071 Granada, Spain; ampmolin@ugr.es; 2 Department of Civil Engineering, University of Granada, 18071 Granada, Spain; ovidio@ugr.es

**Keywords:** fuel tanks, fuel level, air-fuel-water interfaces, plasmonic sensor, surface plasmon resonance

## Abstract

A standard problem in large tanks at oil refineries and petrol stations is that water and fuel usually occupy the same tank. This is undesirable and causes problems such as corrosion in the tanks. Normally, the water level in tanks is unknown, with the problems that this entails. We propose herein a method based on surface plasmon resonance (SPR) to detect in real time the interfaces in a tank which can simultaneously contain water, gasoline (or diesel) and air. The plasmonic sensor is composed of a hemispherical glass prism, a magnesium fluoride layer, and a gold layer. We have optimized the structural parameters of the sensor from the theoretical modeling of the reflectance curve. The sensor detects water-fuel and fuel-air interfaces and measures the level of each liquid in real time. This sensor is recommended for inflammable liquids because inside the tank there are no electrical or electronic signals which could cause explosions. The sensor proposed has a sensitivity of between 1.2 and 3.5 RIU^−1^ and a resolution of between 5.7 × 10^−4^ and 16.5 × 10^−4^ RIU.

## 1. Introduction

The presence of water inside fuel tanks currently poses a problem. The water can originate from condensation and, in regions prone to floods, there is also a higher risk of water infiltrating in the tanks. Also, the water combines with sulfur and other chemical components of the fuel to corrode the inside of the tank [1]. Being denser than the fuel, the water lies at the bottom of the tank, and when the water surpasses the maximum permitted level, it must be removed from the tank.

Currently, it is therefore essential in the fuel industry to have methods to detect the presence of water in fuel tanks and furthermore measure the water level in real time, but fuel tanks in refineries and petrol stations normally use rudimentary methods—for instance, a stick with a special paste (water-finding paste) is inserted into the tank (if the tank is not very deep). When in contact with water, this paste changes color, indicating the presence but not the level of the water in the tank [2].

Different types of sensors have been proposed to measure the level of fuel in tanks, such as sensors based on ultrasonic Lamb waves [3], capacitive sensors [4], pressure sensors [5], and sensors based on optical fiber [6,7]. All these sensors work when a single type of liquid occupies the tank. However, when the tank contains fuel and water, other methods have been proposed to detect the water content in the tank and to measure the levels of the water and fuel in the tank. These methods are based on acoustics [8], microwave reflection [1], reflectometry [9,10], electrode arrays [11], magnetic floats [12], pressure sensors [13,14], and capacitance sensors [15]. All these methods have advantages and disadvantages [16,17].

In the present paper, we have designed an optical sensor that indicates, in real time, the level of air, the level of water, and the level of the gasoline or diesel in the tank. Our sensor is based on surface plasmon resonance (SPR) and overcomes many of the limitations of the sensors proposed to date. It is a safe and rapid-response device that can be used in inflammable or explosive environments such as fuel tanks, as opposed to capacitance sensors or those based on electrodes. Our sensor does not contain movable parts, as opposed to sensors based on floats or pressure sensors, which can become obstructed and are susceptible to mechanical damage. Our device has no problems of friction, heating, or hysteresis, nor does it have the drawback of susceptibility to acoustic or electromagnetic interference.

## 2. Design of the Plasmonic Sensor

The operational principle is based on surface plasmon resonance. Noble metals have a dense assembly of negatively charged free electrons in an equally charged positive-ion background. If an external optical field is applied at one point in the metal, the local density of free electrons at that place in the metal changes due to the force of the field applied. A metal-dielectric interface supports surface plasma oscillations, which are charge-density oscillations (free electrons) along the metal-dielectric interface. Surface plasmon (SP) is the quantum of these oscillations. The SPs are associated with a longitudinal electric field (TM-polarized or p-polarized) that has its maximum at the metal-dielectric interface itself and decays exponentially both on the metal as well as on the dielectric medium.

For SPs to be excited, the condition of resonance must be fulfilled, according to which the wave-vector of the excitation light along the metal-dielectric interface should be equal to that of SPs. One way of achieving this is to excite SPs by an evanescent wave, using a configuration based on a prism. In this case, the resonance condition is given by the following expression [18]:

(1) $$\sin\theta =\sqrt{\frac{\varepsilon_{m}\varepsilon_{s}}{\varepsilon_{p}\left(\varepsilon_{m}+\varepsilon_{s}\right)}}$$

where ε*_m_*, ε*_s_*, ε*_p_* are the dielectric constants of the metal, the medium and the hemispherical prism, respectively, and θ is the angle of incidence respect to the normal on the prism base. Under resonance conditions, the energy of the incident light is transferred to the SPs, resulting in a sharp dip in the intensity of the light reflected at the interface of the prism base and the medium in contact. This occurs at an angle greater than the critical angle.

**Figure 1:**
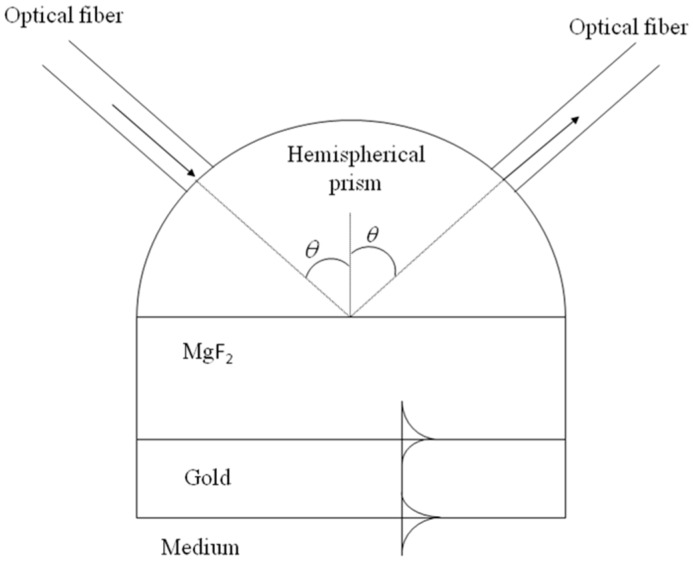
Scheme of the plasmonic sensor. On the left part of the hemispherical prism, the optical fiber transports the incident radiation and on the right, another optical fiber collects the reflected radiation. Also, the surface plasmon polaritons (SPP) are shown propagating through the MgF_2_-gold and gold-medium interfaces.

Figure 1 shows the plasmonic sensor that we propose, based on the Kretschmann configuration [19]. It is formed by a hemispherical prism of SF 10 glass (n=1.7231) [20]. At the base of the prism is a layer of magnesium fluoride (MgF_2_, n=1.38 [21]) and then a layer of gold (n=0.12517+3.3326i) [22]. The gold layer is the one in contact with the medium (air, water, gasoline or diesel); we used gold as the outer layer because of its chemical stability. A p-polarized laser beam with a wavelength of 632.8 nm strikes the prism with a normal incidence to the hemispherical prism surface by an optical fiber with its endface adhered to the prism surface. The angle of incidence with respect to the normal in the MgF_2_ is θ (>critical angle). Finally, the light reflected at the prisma-MgF_2_ interface is received by another optical fiber and conducted to an optical power meter.

When the light strikes the hemispherical prism-MgF_2_ interface at an angle greater than the critical angle, an evanescent wave is generated and is propagated along the hemispherical prism-MgF_2_ interface. This evanescent wave could excite two surface plasmon polaritons (SPPs) depending on the thickness of the layers.

The sensor that we propose is based on intensity interrogation. This has a great advantage: as opposed to sensors based on angular interrogation, our sensor does not require moving parts. In sensors based on angular interrogation, when the medium changes (and therefore the refraction index), the resonance angle also changes. Therefore, in these types of sensors, when the medium changes, it is necessary to adjust the incidence angle of the laser beam and move the detector to locate the corresponding angle to the minimum in the reflectance curve. In the case of our sensor, this operates by the modulation in the reflected intensity. The theoretical modeling of SPR reflectance was carried out by using transfer-matrix method to solve the Fresnel equations for the multilayer stack [23,24] with WinSpall software package. We designed the sensor in such a way that when the sensor is in contact with air, a low reflectance value is registered, when the sensor is in contact with water, a mean reflectance value is registered, and when it is in contact with gasoline or diesel, high reflectance values are registered. For this to happen, the sensor has to work at a fixed angle of the laser beam and the reflected beam at 65.5°.

To have the conditions mentioned above (low reflectance in air, mean reflectance in water, and high reflectance in gasoline/diesel), the thicknesses found for the layers of metal and dielectric were 48 nm for the gold layer and 190 for the MgF_2_ layer. As will be seen in Section 4, with this design, the sensor can reliably distinguish air, water, and different types of gasoline or diesel starting from the reflectance value measured with a detector situated at the end of the optical fiber.

## 3. Gauge Construction

**Figure 2:**
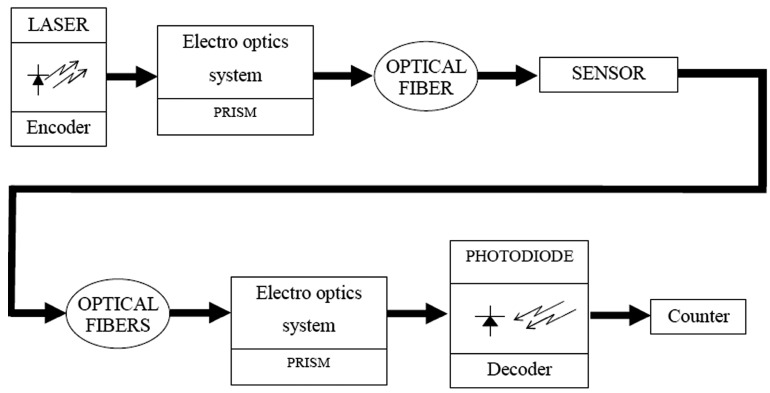
Diagram of the emission-detection system of the device.

The device is composed of two modules. The first part is the module in which the laser beam reaches the sensor (see Figure 2). The encoded radiation from a laser of wavelength of 632.8 nm is incident on a transparent material to which a voltage is applied.

**Figure 3:**
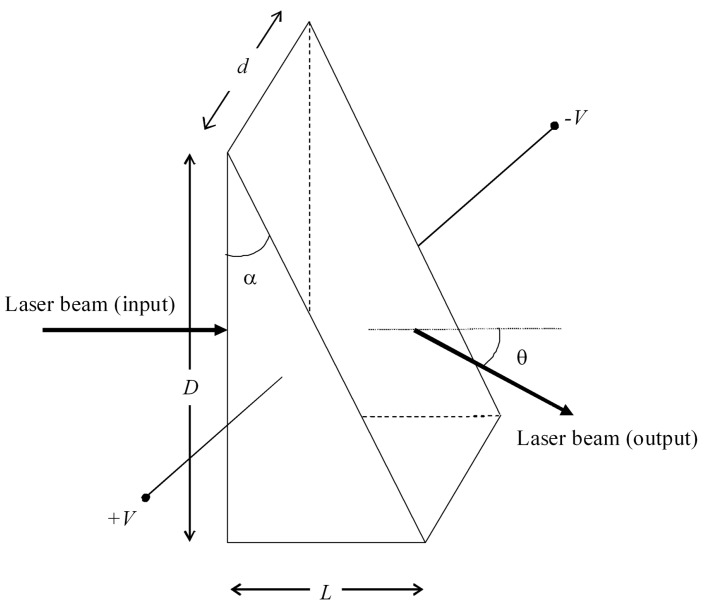
In the electro-optic prism, the deflection angle θ is controlled by the application of a voltage.

**Figure 4:**
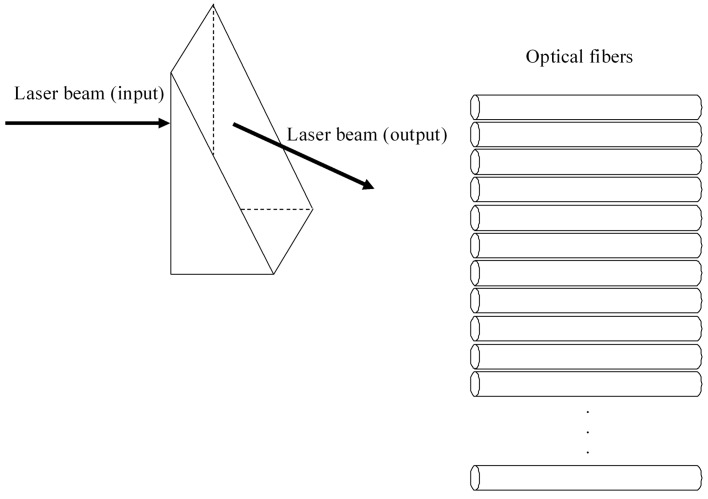
Scheme of the laser beam entering sequentially all the optical fibers. Output endface of each optical fiber is glued to a plasmonic sensor.

In the first stage, the light from the laser is injected in one only optical fiber. With the electro-optical prism we get the same signal from this optical fiber injected into all the necessary optical fiber (see Figure 3 and Figure 4) so that the laser beam exiting the electro-optic prism is sequentially redirected to each optical fiber which transports the light to each plasmonic sensor (Figure 4). An array of plasmonic sensors is located inside the tank. The entrance optical fiber of each plasmonic sensor is glued to a hemispherical miniprism. The entry of light in each hemispherical miniprism is perpendicular (Figure 1). The angle between normal to the base of each hemispherical miniprism and the direction of the light within each hemispherical miniprism has to be 65.5°, as commented in the previous section (Figure 1).

In the second stage (see Figure 2), the laser beam exiting each sensor of the array is guided through each optical fiber. The process of the light path is similar to the path of the illumination system. Now we have a bundle of optical fibers, as many as minisensors (hemispherical prisms) and the light from each optical fiber has to be sequentially injected into only one (Figure 4 with light in opposite direction). The path of the light is the opposite of the stage before, but now the end of the path is the photodiode instead of the sensor.

By means of an electro-optical prism, we inject the light from each optical fiber into only one and, from there, into the photodiode. Both electro-optical prisms work with the same clock signal to synchronize the light signal in the entrance and exit stages. After the photodiode, there is a counter to identify each sensor of the array. The first light emission corresponds to the first sensor, the second emission to the second sensor, and so on. As we know the position of each sensor of the array, we can determine the height of each liquid or level of the air in the tank.

The optical fiber is used to conduct light from the laser to the hemispherical prism and from there to the photodetector. The essential reason is that the optical fiber is not attacked by any of the liquids used, and it transports light instead of electrical signals to avoid any possible explosion in the fuel tanks. The working principle of the electro-optical prism is as follows:

The laser beam can be deflected dynamically by using a prism with an electrically controlled refractive index. The angle of deflection introduced by a prism of small apex angle α and refractive index n is θ≈(n−1)α (see Figure 3). An incremental change of the refractive index Δn caused by an applied electric field E corresponds to an incremental change of the deflection angle [25],

(2) $$\Delta \theta =\alpha \Delta n=\frac{1}{2}\alpha rn^{3}E=\frac{1}{2}\alpha rn^{3}\frac{V}{d}$$

where *r* is the Pockels coefficient or the linear electro-optic coefficient, *n* is the refractive index of the material, *E* the applied electric field, *V* the applied voltage to the material and *d* the prism width. Depending on the maximum deflection angle required, two or more prisms can be cascaded to increase this angle.

An optical beam of width *D* and wavelength λ, has an angular divergence:

(3) $$\delta \theta \approx \frac{\lambda }{D}$$

To minimize that angle, the beam should be as wide as possible, ideally covering the entire width of the prism itself. For a given maximum voltage *V* corresponding to a scanned angle Δθ, the number of independent spots (resolution) is given by:

(4) $$N\approx \frac{\left|\Delta \theta \right|}{\delta \theta }=\frac{\frac{\frac{1}{2}\alpha rn^{3}V}{d}}{\frac{\lambda }{D}}$$

taking into account that:

(5) $$\alpha \approx \frac{L}{D}$$

and the half-wave voltage (the applied voltage necessary to get a phase retardation π) is:

(6) $$V_{\pi }=\left(\frac{d}{L}\right)\left(\frac{\lambda }{rn^{3}}\right)$$

substituting Equations (4) and (5) in Equation (6), we get:

(7) $$N\approx \frac{V}{2V_{\pi }}$$

Therefore, the voltage that we have to apply to the prism is given by [25]:

(8) $$V\approx 2NV_{\pi }$$

where *N* is the number of optical fibers to illuminate (see Figure 4).

The second electro-optical prism works in the same way, but the light comes from the multiplicity of optical fibers to the prism (Figure 4 with the light in the opposite direction) and from it to a single optical fiber and from there to the photodiode.

If the tank is open and the ambient illumination changes, radiation from the exterior could enter the optical fibers. If the illumination conditions change, the radiation in the optical fibers changes; in this case, we could have a variable beam in the optical fiber and therefore a different signal in the output of the photodiode for the same entry signal of the laser beam [26]. For this reason, the laser beam is encoded and the photodiode has to decode the signal. If external radiation enters the optical fibers, the photodiode disregards it, so that the only radiation considered in the photodiode is the beam exiting of the optical fibers. The laser beams of all optical fibers are incident on a single photodiode.

The final signal (height of each liquid or air level) is analyzed by a computer from which the sensor is controlled. These levels are selected by the operator. The alarms can be sounds or visual keys for the blind or deaf, to warn of a dangerous situation. Furthermore, the data can be sent by Internet in real time to a remote point so that the tank can be controlled at all times regardless of its physical location.

The weakest part of the device might appear to be the electro-optical prisms, but these are parts of optical communications and are in fact not weak. All the parts of the optical device, except one part of the optical fiber are outside the tank and it can be sealed in a hermetic box to avoid being broken.

The optical fiber is glued to the minisensors but currently this special glue is extremely strong, so that there are no problems in the sense that the optical fiber could come unglued. The minisensors in the tanks could be housed inside a cylinder (in the tank), for instance, to protect them when the fuel is poured into the tanks. This cylinder has to be open at the same level of the tank.

The coating process is as follows: the base of the hemispherical prism is firstly cleaned with a solution consisting of ethanol and diethyl ether at a 1:1 ratio, rinsed with deionized water, and then dried with nitrogen. The substrate is sequentially coated with a 190 nm MgF_2_ layer and a 48 nm gold layer to construct the sensor chip. The gold layer is coated using magnetron sputtering with the layer thickness measured by a quartz crystal oscillator thickness monitor. The substrate-heating and bias-voltage techniques are used in the coating process to improve the uniformity and firmness of the gold layer thickness. The MgF_2_ layer is deposited using evaporation coating with the layer thickness measured by a step profiler. The MgF_2_ crystals are used as the evaporation materials and the weight is controlled for by the specific layer thickness in the coating process [27].

## 4. Results and Discussion

**Figure 5:**
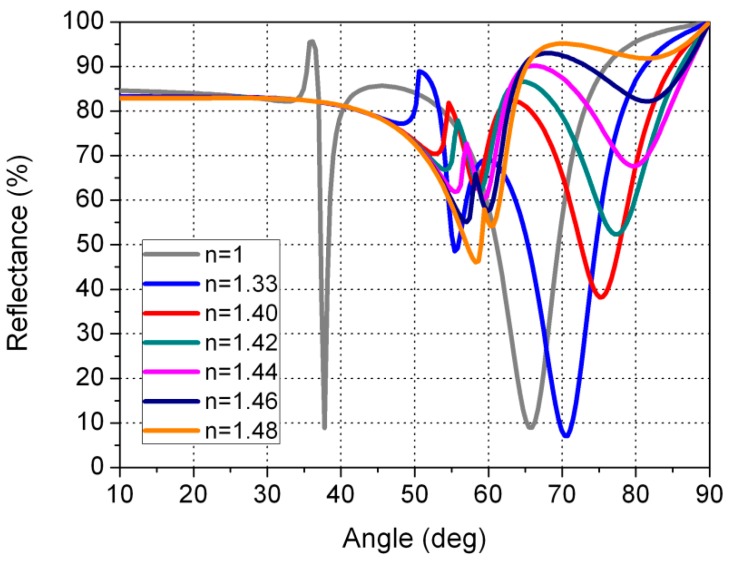
Reflectance curves as a function of the angle of incidence of the light in the prism. These represent the curves when the sensor is in contact with air (n=1), water (n=1.33), and gasoline or diesel fuel (n=1.40−1.48). The thickness of the gold layer is 48 nm, and the thickness of dielectric layer of the MgF_2_ is 190 nm.

Figure 5 shows the reflectance registered by a photodiode as a function of the angle of incidence of the light in the MgF_2_ layer. Figure 5 shows the reflectance curves when the sensor is in contact with air (n=1), with water (n=1.33), and with gasoline or diesel. For the refractive index of gasoline and diesel, a range was taken of between 1.40 and 1.48, which corresponds to the values for different types of gasoline and diesel [28,29,30,31,32,33]. As commented in Section 2, the sensor that we propose (see Figure 1) is based on the intensity interrogation method, which uses a dielectric MgF_2_ layer to excite two plasmons. These surface plasmon resonances can be clearly seen as two minimums in the reflectance when the sensor is in contact with air. In this case, the reflectance dip at 37.7° is associated with the air/gold SPP, while the dip at 65.5° with the MgF_2_/gold one. Our sensor works at a fixed angle of 65.5°, the angle of incidence in the MgF_2_ layer. When the medium in contact with the sensor changes, the resonance characteristics change also, altering the shape of the reflectance curves.

The values of the refractive indices of the gasolines and diesels measured in our laboratories are 1.43 and 1.42 for Efitec 95 Neotech and Efitec 98 Neotech gasoline, respectively, and 1.46 for the two types of diesel (e+ Neotech diesel and e + 10 Neotech diesel). The four fuels are from the REPSOL company. The measurements were made with an Phywe 62,409.00 optical refractometer (PHYWE Systeme GmbH & Co. KG, Göttingen, Germany.). We have also measured the refractive-index values for 589.3 nm and 632.8 nm by minimum-deviation methods using an spectrogoniometer, and the results varied only in the third decimal of the refractive index, as reported by other authors [34,35]. As can be seen, the experimental measurements of our gasolines and diesels are within the range of values for which we have made the calculations.

**Figure 6:**
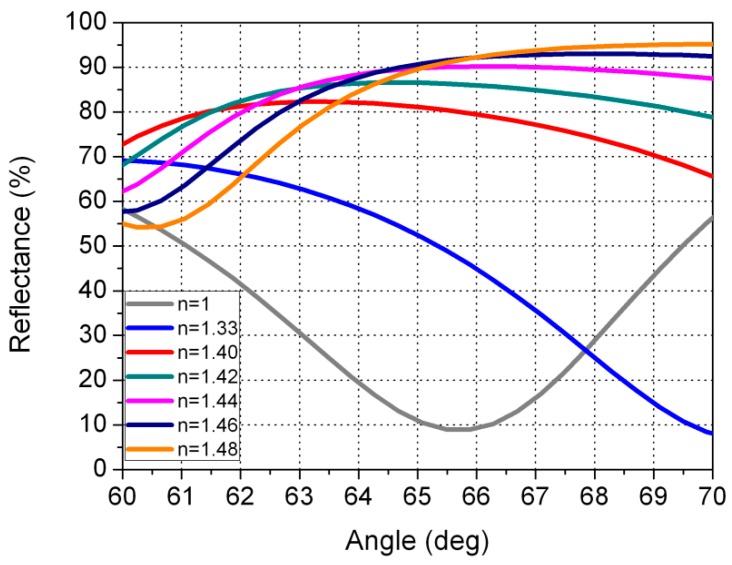
Detail of Figure 6. Reflectance curves from Figure 5 for incidence angles of between 60° and 70°.

As reflected in Figure 6 (detail from Figure 5 for an angle range of between 60° and 70°, where the angle of interest is shown), when the sensor is in contact with air a reflectance value of 9.0% is registered; when it is in contact with water, the value is 49.1%; and when it is in contact with gasoline or diesel the values are 80.4%, 86.4%, 90.0%, 91.5%, and 91.0% for refractive-index values of 1.40, 1.42, 1.44, 1.46, and 1.48, respectively. It is important to note that our sensor provides reflectance values above 80% for gasoline and diesel. In this way, the sensor in the tank continuously distinguishes between air, water, and gasoline or diesel, since when the reflectance measured with the photodiode is lower than 10%, the sensor that is in this position would be indicating that in this position corresponds to air. If a given sensor in the tank provided a reflectance of around 49.1%, this would indicate that there would be water. Finally, if the sensor provided a reflectance higher than 80%, this would indicate gasoline or diesel.

**Figure 7:**
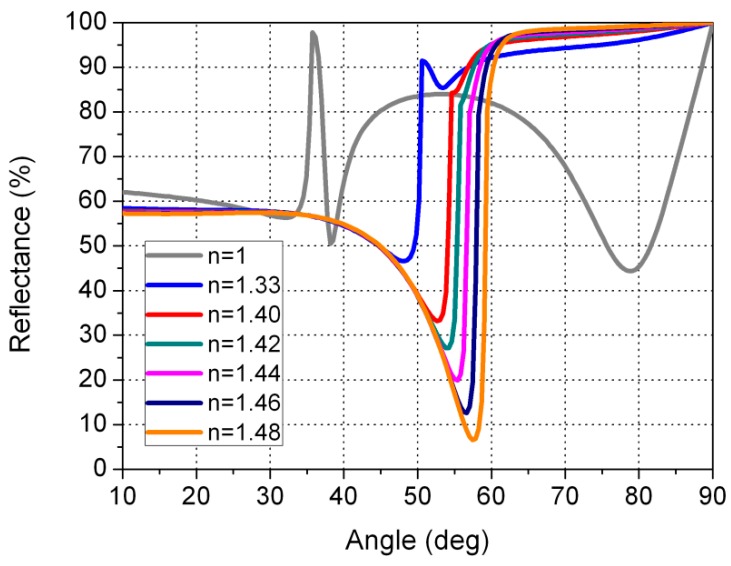
Reflectance curves as a function of the angle of incidence of the light in the prism for a gold layer of 28 nm. The thickness of the dielectric layer of the MgF_2_ is 190 nm.

**Figure 8:**
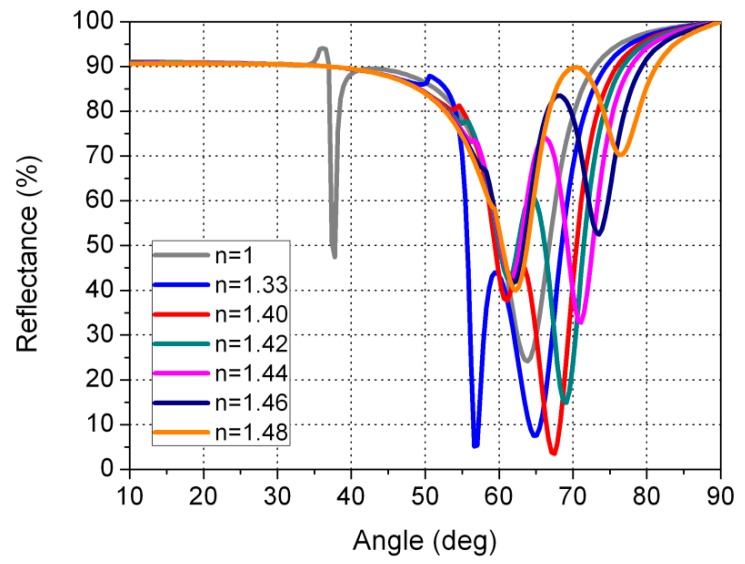
Reflectance curves as a function of the angle of incidence of the light in the prism for a gold layer of 68 nm. The thickness of the dielectric layer of the MgF_2_ is 190 nm.

We have also made the calculations for other thicknesses (28 and 68 nm). As can be seen in Figure 7, when the sensor is in contact with water, the reflectance is very close to the reflectance when the sensor is in contact with gasoline or diesel. This could cause the sensor to fail to distinguish reliably between water and gasoline or diesel. A similar failure occurs for gold layer thicknesses greater than 48 nm. In this case, (Figure 8), if we consider the angle of 75°, the reflectance curves for air, water, and gasoline/diesel are very close together. Therefore, we designed the sensor in such a way that the reflectance for air is low (9.0%), medium for water (49.1%), and high for gasoline/diesel (>80.4%). We achieved this for a gold thickness of 48nm. In this way, the sensor can easily distinguish water from gasoline/diesel.

Tanks in petrol stations usually have a capacity of 20,000–50,000 liters stored in a cylinder. The inner diameter of the base of the cylinder is approximately 2.5 m. The cylindrical tanks are placed parallel to the ground to avoid deep excavation, so that the height of the sensor would be 2.5 m.

The critical height of water in petrol station tanks is approximately 15 cm. If a hemispherical prism is located every 2 cm, in this case, the accuracy of the measurement of the interface position would be high but we would have to use 125 hemispherical prisms; however, if we locate one every 5 cm, we would have less accuracy but could use only 50 hemispherical prisms. Even with an accuracy of 5 cm, we would know whether the height of water is the adequate or not. In practice, we must reach a compromise between accuracy and cost.

One drawback of the sensors based on wavelength interrogation is that they require a spectrometer to analyze the light spectrum at the exit of the sensor [36,37]. In the case of our sensor, we would need 50 spectrometers or 125 spectrometers (depending on the accuracy of the measurement of the interface position mentioned above) to analyze the light spectrum leaving each optical fiber. Such a large quantity of spectrometers would enormously increase the complexity, size, and cost of the sensor.

In addition, spectrometers need a certain amount of time to measure a spectrum. For example, Coelho *et al.* [37] used an optical spectrum analyzer with a resolution of 0.2 nm and a sampling rate of 1 spectrum *per* min, and a BraggMETER FS2200SA interrogator (eTester, Verona, VA, USA)) with a resolution of 1 pm at a sampling rate of 1 spectrum *pe*r s. Considering that our sensor works by sequentially redirecting the laser beam exiting the electro-optic prism to each optical fiber, our sensor would work more slowly if it were based on wavelength interrogation due to the time spent by the spectrometers for analyzing the output spectrum of each fiber.

Also, on some occasions, it is necessary to apply a special treatment of the signal in sensors based on wavelength interrogation, as for example applying fast Fourier transform smoothing filters to the spectral data to reduce the noise due to the low power level of the light that reaches the spectrometer [37]. This type of signal processing or similar ones would also slow down and complicate the functioning of our sensor.

In short, we have designed our sensor based on intensity interrogation because if it were based on wavelength interrogation the sensor would be not only more complex, but bigger, slower, and more expensive too.

The sensitivity of our sensor can be calculated from the change in reflectance *per* unit of change of refractive index. With respect to gasolines and diesels, we have considered the mean value of the refractive index (1.44) and the mean reflectance value (0.879), since the reflectance for gasolines and diesels does not vary linearly with the refractive index. Therefore, to calculate the sensitivity, we have considered 1, 1.33, and 1.44 for the refractive index and 0.09, 0.491, and 0.879 for the reflectance. Considering two consecutive values, we get a sensitivity of 1.2 and 3.5 RIU^−1^, where RIU indicates the units of the refractive index.

The sensor resolution depends upon the accuracy with which the monitored SPR parameter can be determined by the specific sensing device and as such is limited by sensor-system noise [38]. To calculate the resolution of the sensor, we divide the accuracy of the photodetector by its sensitivity. Considering an accuracy of 0.2% in the signal registered by the photodetector [38,39], we get a resolution of 16.5 × 10^−4^ and 5.7 × 10^−4^. Below, we compare our sensor, in terms of sensitivity and resolution, with other proposed in the literature.

Lin *et al.* [40] report sensitivity values of 3.8 and 4.16 RIU^−1^ for a tapered optical-fiber sensor based on localized surface plasmon resonance with an operating range of 0.07 RIU. The resolutions are 3.7 × 10^−5^ and 3.2 × 10^−5^ RIU, considering a standard deviation of noise of the sensor output of 1.42 × 10^−4^ and 1.35 × 10^−4^, while in our case we have considered an accuracy of 0.2%. The great operating range of our sensor (0.48 RIU) makes the sensitivity somewhat lower. On the other hand, the resolution is also different, since it depends on the accuracy of the optical signal registered by the photodetection system used.

Chen *et al*. [41] have proposed an optical fiber biosensor based on silver nanoparticles, with a sensitivity of 160%/RIU and an operating range of 0.07 RIU. This value is within the range of our sensor (120%/RIU and 350%/RIU). To calculate the resolution, the authors considered a value for the standard deviation of noise of the sensor output of 0.75%, and they got a resolution of 4.68 × 10^−3^ RIU. Considering the same value of 0.75%, we get resolutions of 2.1 × 10^−3^ and 6.2 × 10^−3^ RIU for our sensor.

Wang *et al.* [42] report a resolution of 2 × 10^−4^ RIU for an optical fiber sensor based on Kretschmann’s configuration. The operating range of their sensor is 0.025 RIU and they consider an accuracy of 0.1% in the signal registered. If we use 0.1% for the calculation of the sensitivity of our sensor, we get resolutions of 2.8 × 10^−4^ and 8.2 × 10^−4^ RIU.

We can also compare the sensitivity of our sensor with the sensitivity of the sensors based on the wavelength interrogation. For example, Liu *et al.* [36] present a surface plasmon resonance sensor based on silver-coated hollow-fiber structure for the detection of liquids with high refractive index. The operating range of this sensor is 0.06 RIU and is based on wavelength interrogation. This sensor has a resolution of between 0.8 × 10^−4^ and 5.1 × 10^−4^ RIU.

Coelho *et al.* [37] have proposed two hybrid sensors based on a fiber Bragg grating for monitoring organic solvents in high-refractive-index edible oils. With the configuration in intensity interrogation, they get a resolution of 2.1 × 10^−4^ RIU for one of them and 2.3 × 10^−4^ RIU for the other in transmission mode and 1.4 × 10^−4^ RIU in the reflection mode. The first of the sensors is also interrogated in wavelength, providing a resolution of 5.8 × 10^−4^ RIU in transmission mode.

As indicated, our sensor has a sensitivity and a resolution similar to those of other sensors based on intensity interrogation, and even those based on wavelength interrogation. In general, sensors based on wavelength or angular interrogation have greater sensitivity and resolution than those based on intensity interrogation [38]. However, our sensor does not require a high resolution, given that the aim of the sensor proposed is simply to distinguish between air, water, and gasolines/diesels. Also, taking into account that we do not need to distinguish between different types of gasoline/diesel (refractive index between 1.40 and 1.48), a resolution of 10^−2^ RIU would be sufficient to distinguish between media with indexes of 1, 1.33, and 1.40–1.48. Finally, with respect to the sensors based on angular interrogation, the advantage of our sensor is that it does not require moving parts, as mentioned in Section 2.

## 5. Conclusions

Here, we present an optical sensor for fuel tanks based on surface plasmon resonance. The sensor detects not only water-fuel and fuel-air interfaces, but can also measure the level of every liquid contained in the tank in real time. A major advantage is that this sensor can measure the height of the fuel (gasoline or diesel) without distinguishing between them, whereas other sensors have to be specific for gasoline or diesel.

This sensor serves for inflammable liquids such as gasoline, diesel, other crude-oil derivatives, acetones, alcohols, *etc.* because there are no electrical or electronic signals inside the tank but only light radiation. This makes it impossible to generate electrical sparks that could cause explosions. The laser and the photodetector, which work with electronic circuits, remain outside the tank.

As we are encoding light emitted by the laser, the influence of any external illumination that could enter the tank is avoided. Therefore, the device measures the height of several liquid levels in any type of tank, whether opaque, translucent, or transparent.

In addition, the device continuously measures the levels of all kinds of liquids that do not attack the optical fiber, hemispherical prism, or the plasmonic structures used.

With respect to the light-emission system and the photodetection system, our device uses only one laser and only one photodiode. Only one laser illuminates all the optical fibers and only one photodetector receives the radiation from all the optical fibers.

By knowing the tank geometry and the spacing of the sensors, we can track the volume (in real time) of the fuel and water in the tank, and therefore we can also calculate the flow rate, *i.e.*, how much liquid enters and leaves the tank per time unit.

The overall system is equipped with a visual and audible alarm that can be regulated to any liquid height. Also, the gauge is controlled by a PC that can store the data, print them or send them in real time by Internet worldwide. Finally, it is not necessary to carry out periodic calibrations.

## Abbreviations

The following abbreviations are used in this manuscript:

SPP	Surface plasmon polaritons
SPR	Surface plasmon resonance
TM	Transversal magnetic
